# BECN1 mRNA expression in breast cancer tissue; significant correlation to tumor grade

**DOI:** 10.1007/s00438-024-02145-2

**Published:** 2024-05-24

**Authors:** Sarah Ahmed Aglan, Ahmed Mostafa Awad, Yasmine Nagy Elwany, Sanaa Shawky, Radwa Mohamed Abdel Salam, Rasha Said Omar, Rasha Abdel Mawla Ghazala, Nada Ahmed Soliman, Marwa Ibrahim Khedr, Lamia Said Kandil, Mohamed Sultan, Yasser Hamed, Noha Said Kandil

**Affiliations:** 1 Department of Chemical Pathology, Medical Research Institute, Alexandria University, Alexandria, Egypt; 2 Department of Cancer Management and Research, Medical Research Institute, Alexandria University, Alexandria, Egypt; 3 Department of Pathology, Medical Research Institute, Alexandria University, Alexandria, Egypt; 4 Department of Biochemistry, faculty of medicine, Alexandria University, Alexandria, Egypt; 5 School of Pharmacy and Biomedical sciences, University of central Lancashire, Preston, UK; 6 Department of Experimental and clinical surgery department, Medical Research Institute, Alexandria University, Alexandria, Egypt

**Keywords:** Breast cancer, Beclin 1, Autophagy, DFS

## Abstract

Breast cancer (BC) is a heterogenous disease with multiple pathways implicated in its development, progression, and drug resistance. Autophagy, a cellular process responsible for self-digestion of damaged organelles, had been recognized as eminent player in cancer progression and chemotherapeutic resistance. The haploinsufficiency of Beclin 1 (BECN1), autophagy protein, is believed to contribute to cancer pathogenesis and progression. In our study, we investigated the expression of BECN1 in a BC female Egyptian patient cohort, as well as its prognostic role through evaluating its association with disease free survival (DFS) after 2 years follow up and association of tumor clinicopathological features. Twenty frozen female BC tissue samples and 17 adjacent normal tissue were included and examined for the expression levels of BECN1. Although the tumor tissues showed lower expression 0.73 (0–8.95) than their corresponding normal tissues 1.02 (0.04–19.59), it was not statistically significant, p: 0.463. BECN1 expression was not associated with stage, nodal metastasis or tumor size, p:0.435, 0.541, 0.296, respectively. However, statistically significant negative correlation was found between grade and BECN1 mRNA expression in the studied cases, p:0.028. BECN1 expression had no statistically significant association with DFS, *P* = 0.944. However, we observed that triple negative (TNBC) cases had significantly lower DFS rate than luminal BC patients, p: 0.022, with mean DFS 19.0 months, while luminal BC patients had mean DFS of 23.41 months. Our study highlights the potential role of BECN1 in BC pathogenesis, showing that BECN1 expression correlates with poorer differentiation of BC, indicating its probable link with disease aggressiveness. DFS two years follow up showed that TNBC subtype remains associated with less favorable prognosis.

## Introduction

Female breast cancer, currently the highest incidence of diagnosis among all cancers accounting for more than 11% of newly diagnosed cases, surpassing lung cancer, according to GLOBOCAN 2020 report (Sung et al. 2021). Breast cancer in Egypt, despite its low incidence figures, represents the most commonly diagnosed cancer in females mirroring the global data. However, its mortality rate surpasses worldwide data, nearly the double, owing to the late diagnosis and refrainment from screening. Moreover, a striking finding in Azim et al. metanalysis 2023 was the high frequency of young age of presentation in Egypt which alone is a bad prognostic factor. (Azim et al. 2023) While late diagnosis can be overcome by intense screening programs and raising awareness, the young age of presentation warrants further investigation of the disease behavior, genetic analysis, environmental studies as well as revisiting and evaluation of screening strategies.

Breast cancer is a heterogenous disease of complex molecular basis that is yet to be unraveled. Chemoresistance is a great challenge also, thus understanding its molecular basis and possible culprits is crucial for proper disease management (Feng et al. 2018). Autophagy, a cellular adaptive process occurs in response to metabolic stress. It involves self-digestion through lysosomal degradation of damaged organelles as well as other threats including pathogens and misfolded proteins. The digested components can be recycled and used in energy production. Autophagy is regarded as a quality control for cellular organelles working hand in hand with ubiquitin proteasome degradation machinery maintaining cell survival. They act through prevention of accumulation of protein aggregates, which might cause oxidative stress leading to genomic damage, mutagenesis and cancer promotion. However, if unrestrained, autophagy can lead to progressive cellular destruction and eventually cell death (Mathew et al. 2007).

There are three types of autophagy; “macro autophagy, micro autophagy and chaperone- mediated” (Aman et al. 2021). Macro autophagy (referred hereafter to as autophagy) is a multistep process that results in the formation of “Autophagosome”; a double membrane structure formed by engulfment of cytoplasm by a “phagophore/ isolation membrane”. The phagophore is formed by initiation process involving phosphorylation of multiple proteins by Unc51-like kinase complex (ULK complex). Further nucleation occurs mediated through phosphatidylinositol 3-kinase class III (PIK3C3) complex involving Beclin 1 (BECN1) and PIK3C3/VPS 34 (Vacuolar protein sorting 34) genes among others. Elongation and fusion of the phagosome membranes follows engulfing target cargo and end in the formation of autophagosomes which eventually fuses with lysosomes and results in the degradation of its content (Li et al. 2010; Mizushima 2011; Vega-Rubín-de-Celis 2019).

Beclin 1 (BECN1) gene is located on chromosome 17q21 encoding for a protein involved in autophagy pathway as aforementioned. It is regarded as a tumor suppressor gene where its haploinsufficiency is considered the main mechanism that is implicated in tumorigenesis rather than mutations occurring in the gene, which are not frequently encountered in cancers (Vega-Rubín-de-Celis 2019). In breast cancer, BECN1 was found to exhibit loss of heterogeneity in 45% of breast cancer tissue, where deletion of one allele is observed. Epigenetic silencing is observed through CpG island methylation in breast cancer tissues as well (Li et al. 2010; Vega-Rubín-de-Celis 2019).

Inhibition of autophagy is currently gaining a lot of interest as a potential promising route through which cancer therapy could be commenced particularly in treating resistant tumors (Lim and Murthy 2020). Of note, resistance to therapies involving kinase inhibitors was attributed to their proposed effect on autophagy, as they share common targets with signaling PI3K/AKT/mTOR (mammalian target of rapamycin) pathway (Amaravadi et al. 2011).

In our study, we aimed at studying BECN1 expression in tumor and adjacent normal tissues as well as correlation to clinicopathological features and disease-free survival after 2 years of follow up.

## Methods

Twenty cases of primary breast cancer were included in the study. Patients enrolled in our study were newly diagnosed with BC, newly diagnosed selection was based upon possible acquisition of fresh tissue for maximum mRNA recovery from samples. No selection bias was imposed or exclusion of patients, however, patients suffering from distant metastasis, any other malignancy or receiving radiotherapy were excluded. Fresh tissue samples from the mass as well as from adjacent normal breast tissues were taken and subjected immediately to freezing at -80◦C. Normal breast tissue samples were available for 17 cases. Similar samples were obtained and fixed in 10% formaldehyde for subsequent routine H&E (Hematoxylin and Eosin) stain as well as immunohistochemistry (IHC) for Estrogen receptor (ER), Progesterone receptor (PR) and Human epidermal growth factor receptor 2 (HER2).

Consents were taken from patients under ethical approval of the Ethical Committee of the Medical Research Institute, approval number IORG0008812, and following the Helsinki declaration.

Patients were recruited and followed up from January 2019 to December 2021. Patients included received care at the department of Experimental and Clinical Surgery, Medical Research Institute, Alexandria University, Alexandria, Egypt. Patients were all newly diagnosed. Clinicopathological data; tumor size, nodal status, metastatic workup, ER, PR, HER2 expression by immunohistochemical (IHC) analysis, tumor grade and histopathological type, were gathered from records in Pathology department and Cancer Management and Research department of the Medical Research Institute.

### Clinical examination and follow up

Patients were clinically examined in Cancer Management and Research department for TNM staging, treatment and follow up. We followed up all patients for 2 years after presentation for detection of disease relapse defined as time between surgical removal of the tumor until first local relapse or distant metastasis within 2 years from recruitment.

Treatment regimen after surgery for all was 4 cycles of (doxorubicin 60 mg/m2- cyclophosphamide 600 mg/m2) regimen followed by paclitaxel (Taxane). 3 Patients out of 4 with HER2 positive tumors received Herceptin. Neoadjuvant treatment was offered for 7 patients.

### Pathological examination

The formalin fixed paraffin embedded (FFPE) tissue sections were cut into 4µ thick sections for routine H&E stain and IHC. Histopathologic classification and grading of tumor tissue followed the Nottingham modification of the Bloom–Richardson system (Bloom and Richardson 1957). IHC analysis for ER, PR and HER2 expression was done according to recommendations (Hammond et al. 2010; Wolff et al. 2013). and equivocal results were retested by Fluorescence In Situ Hybridization (FISH) and reclassified accordingly.

### Molecular analysis

#### *Ribonucleic acid (RNA) extraction and reverse transcription

Sections of the fresh tissues obtained were stored frozen in later (RNAlater™ Stabilization Solution, cat no. AM7023), to prevent RNA degradation, in -80^o^C.

RNA extraction was done using RNeasy Mini Kit, Qiagen, Cat. No. / ID: 74,104. Extraction of total RNA for all tissues was done according to manufacturer instructions. RNA quality and quantity for each sample were verified using Nanodrop spectrophotometer (Thermo Scientific, USA). Complementary Deoxyribonucleic acid (cDNA) development using high-capacity cDNA reverse transcription Thermo-Fisher, Cat no. 4,368,814. Protocol and thermal cycle steps and conditions were done according to manufacture’’s instructions.

#### Real time analysis for BECN1 mRNA expression

BECN1 mRNA relative quantitation was done in both tumor and normal tissues using real time PCR analysis (Bio-Rad CFX connect real time PCR instrument) using BECN1 (Hs01007018_m1) probe, Thermo-Fisher (Cat. No.: 4,331,182) and TaqMan universal master mix II, no UNG Thermo-Fisher, Cat no.: 4,440,043. GAPDH (Hs02786624_g1) probe Thermo-Fisher, (Cat. No.: 4,448,489) was used for amplification of Glyceraldehyde-3-phosphate dehydrogenase (GAPDH) mRNA as a reference gene expression for normalization of BECN1 mRNA expression to compensate for variability in RNA extraction and reverse transcription efficiencies and input volumes. In 20 ul reaction tube, cDNA was mixed with the TaqMan universal master mix and probes. Thermal cycle conditions were initial incubation for polymerase activation at 95 ^o^C for 10 min then for 45 cycles; denaturation at 95 ^o^C for 15 s followed by annealing and extension at 60 ^o^C for 60 s. Relative expression of BECN1 was calculated using 2^−∆∆CT^.

### Data analysis

BECN1 mRNA expression was expressed as median and range and its ratio in tumor tissue to adjacent normal one was calculated. We used IBM SPSS software package version 20.0. and 29.0 (IBM Corp). Categorical data were represented as numbers and percentages. For continuous data, they were tested for normality by the Shapiro-Wilk test. Quantitative data were expressed as range (minimum and maximum), mean, standard deviation and median. Mann Whitney test was used to compare two groups for not normally distributed quantitative variables. Wilcoxon signed ranks test was used for abnormally distributed quantitative variables, to compare between tumor and normal tissue expressions of BECN1 mRNAs in the same patient. Kruskal Wallis test was used for abnormally distributed quantitative variables, to compare between more than two studied groups. Spearman coefficient was used to correlate between two distributed abnormally quantitative variables. Kaplan-Meier was used for the relation with disease free survival. Significance of the obtained results was judged at the 5% level. P-value of less than 0.05 was considered statistically significant.

## Results

### Clinicopathological data

**Fig. 1:**
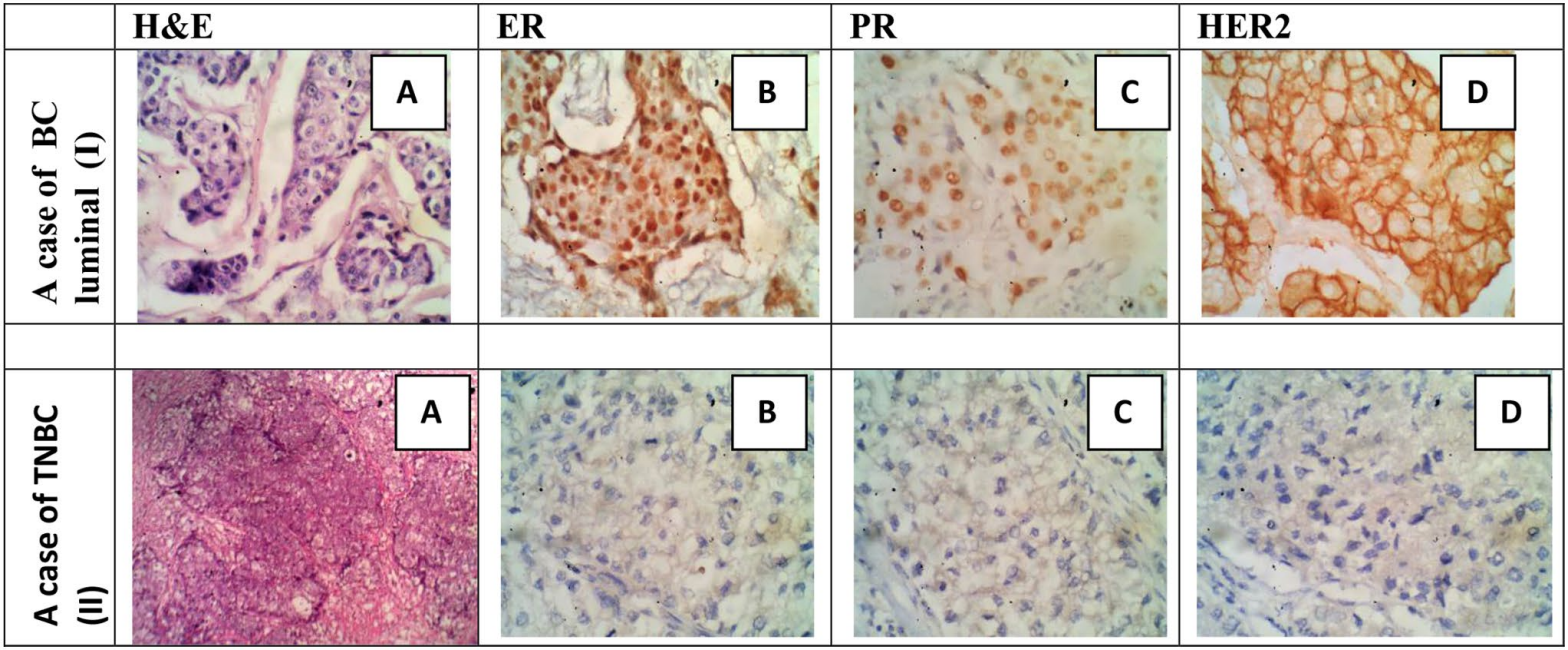
(I) Case of BC luminal. **A**: IDC GII (H&E, x40); **B**: strong positive ER nuclear staining (IHC, x40); **C**: moderate positive PR nuclear staining (IHC, x40); **D**: strong complete membranous HER2 staining (IHC, x40). (II) Case of TNBC. **A**: IDC GIII (H&E, x40); **B**, **C** and **D**: negative staining for ER, PR and HER2 (IHC, x40)

Twenty cases were diagnosed histopathologically as cases of invasive ductal carcinoma (IDC), No special type (NST). 17 cases of IDC grade II showed invasive trabeculae, cords and nests of malignant ductal cells having pleomorphic vesicular nuclei and abundant eosinophilic cytoplasm, surrounded by a desmoplastic stroma. The 3 cases of IDC grade III- showed invasive sheets of malignant ductal cells having highly pleomorphic vesicular nuclei with frequent mitotic fures. Figure 1.

Only one patient had a family history of breast cancer. Patients age median at presentation was 49 ranging from 35 to 71 years old. Further clinical and histopathological features are listed in Table 1.

**Table 1:** Patients’ clinicopathological data (*n* = 20)

	No. (%)
*Age (years)*	
≤ 49	11 (55%)
> 49	9 (45%)
Mean ± SD.	48.92 ± 9.6
Median (Min. – Max.)	49 (35–71)
*Stage*	
I	4 (20%)
II	10 (50%)
III	6 (30%)
*Tumor largest diameter*	
Median (Min. – Max.)	2.6 (1.0–5.8)
Nodal status (positive)	11 (55%)
ER positive	17 (85%)
PR positive	17 (85%)
*HER2*	
Negative	16 (80%)
Overexpressing	4 (20%)
*Type*	
Luminal	17 (85%)
TNBC	3 (15%)
*Tumor grade*	
II	17 (85%)
III	3 (15%)
*DFS at 2 years (in months)*	
Median (Min. – Max.)	24 (13–24)

SD: Standard deviation, DFS: Disease free survival, TNBC: Triple negative breast cancer, ER: Estrogen receptor, PR: Progesterone receptor, HER2: Human epidermal growth factor receptor-2

### Correlation between BECN1 mRNA expression and patients’ age, tumor grade, stage, and size

As shown in Table 2, BECN1 T/N ratio was significantly negatively correlated with grade of tumor. However, no statistically significant correlation was observed with age, tumor stage, or size (largest diameter).

**Table 2:** Correlation between BECN ratio and different parameters

	BECN1 T/*N* ratio	
	*r*_s_	*p*
Age	0.320	0.113
Stage	−0.043	0.435
Tumor grade	−0.473	0.028^*^
Tumor largest diameter	−0.140	0.296

r_s_: Spearman coefficient, p: p- value Correlation is significant at the 0.05 level (1-tailed), *significant result

### BECN1 mRNA relative expression in breast cancer tumor versus adjacent normal tissues

**Fig. 2:**
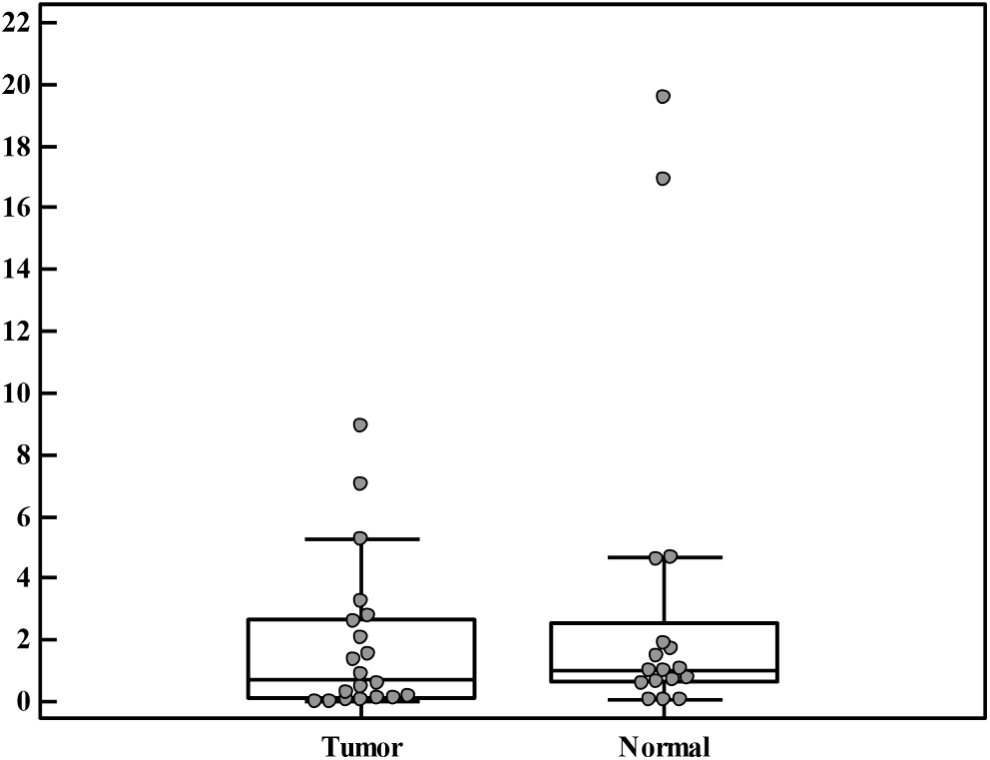
Box-plot graphical representation of the median and range of BECN1 2^−∆∆CT^ (relative fold expression) in tumor and adjacent normal tissues

We analyzed the expression of BECN1 in relation to GAPDH in both tumor tissues and their corresponding adjacent normal tissues. Although the tumor tissues showed lower expression 0.73 (0–8.95) than adjacent normal tissues 1.02 (0.04–19.59), Table 3; Fig. 2, it did not however reach the level of statistical significance, p: 0.463, Table 3. The ratio of BECN1 expression in tumor tissue to adjacent normal tissue (BECN1 T/N ratio) for each case was calculated. The median of ratios was 0.84 (0–111.43), where 10 cases (58.8%) were below 1 and 7 (41.2%) cases were ≥ 1, Table 4.

**Table 3:** Comparison between tumor and adjacent normal tissue according to BECN1/GAPDH expression

	Tumor (*n* = 20)	Normal (*n* = 17)	*p*
*BECN1*			
Median (Min. – Max.)	0.73 (0–8.95)	1.02 (0.04–19.59)	0.463

Statistical analysis was done by Wilcoxon signed ranks test SD: Standard deviation, p: p value for comparing between tumor and normal

**Table 4:** BECN1 T/N ratio: 17 cases

BECN1 T/N ratio (*n* = 17)	
< 1	10 (58.8%)
≥ 1	7 (41.2%)
Median (Min. – Max.)	0.84 (0–111.43)

### Association between BECN1 mRNA expression and clinicopathological criteria

Out of 17 cases used for BECN1 mRNA T/N ratio, 9 had positive nodal metastasis, while 14 cases had luminal type breast cancer and 3 had triple negative type. When BECN1 mRNA expression was analyzed for association with nodal status, ER, PR, HER2 expression as well as molecular subtypes, no statistically significant relation was observed as shown in Table 5.

**Table 5:** Association between BECN1 T/N ratio and different parameters (*n* = 17)

	N	BECN1 T/N ratio	Test of Sig.	P
		Median (Min. – Max.)		
*Nodal status*				
Negative	**8**	0.63 (0.0–12.47)	U = 29.50	0.541
Positive	**9**	0.93 (0.01–111.4)		
*ER*				
Negative	**3**	0.59 (0.42–3.32)	U = 19.0	0.859
Positive	**14**	0.885 (0.0–111.4)		
*PR*				
Negative	**3**	0.59 (0.42–3.32)	U = 19.0	0.859
Positive	**14**	0.885 (0.0–111.4)		
*HER2*				
Negative	**13**	0.84 (0.0–111.4)	U = 23.5	.785
Overexpressing	**4**	0.49 (0.01–5.46)		
*Subtype*				
Luminal	**14**	0.885 (0.0–111.4)	U = 19.0	0.859
TNBC	**3**	0.59 (0.42–3.32)		

SD: Standard deviation U: Mann Whitney test H: H for Kruskal Wallis test

p: p value for relation between BECN ratio and different parameters, TNBC: Triple negative breast cancer, ER: Estrogen receptor, PR: Progesterone receptor, HER2: Human epidermal growth factor receptor-2

### Disease free survival association with BECN1 expression and clinicopathological features of the patients

**Fig. 3:**
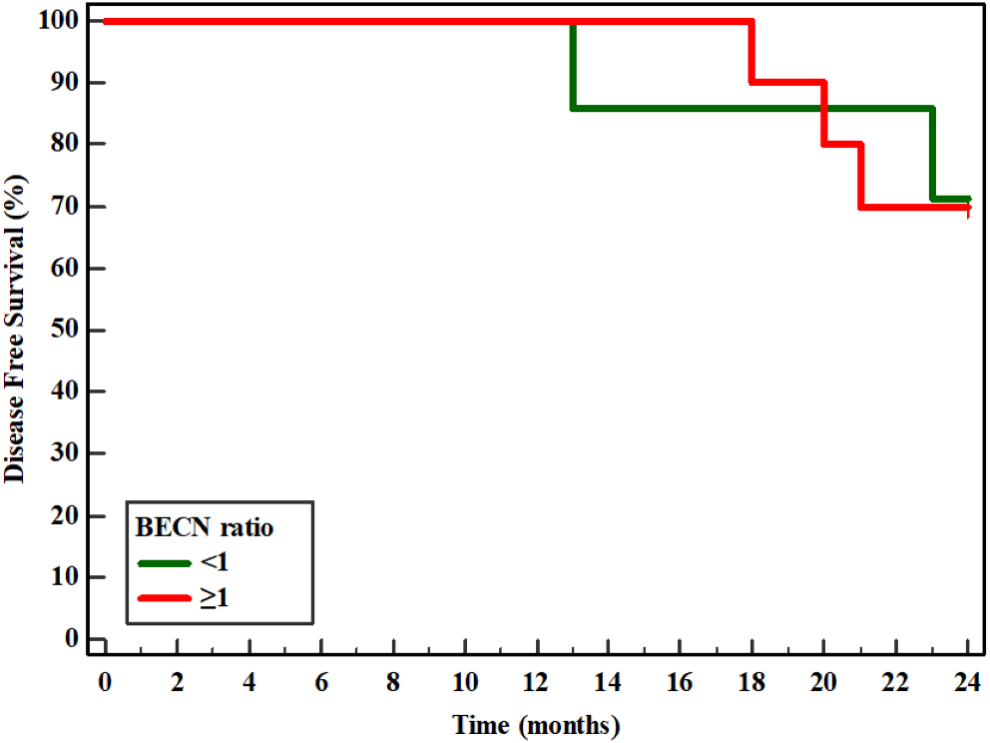
Kaplan-Meier survival curve for Disease Free Survival with BECN1 ratio (*n* = 17)

**Fig. 4:**
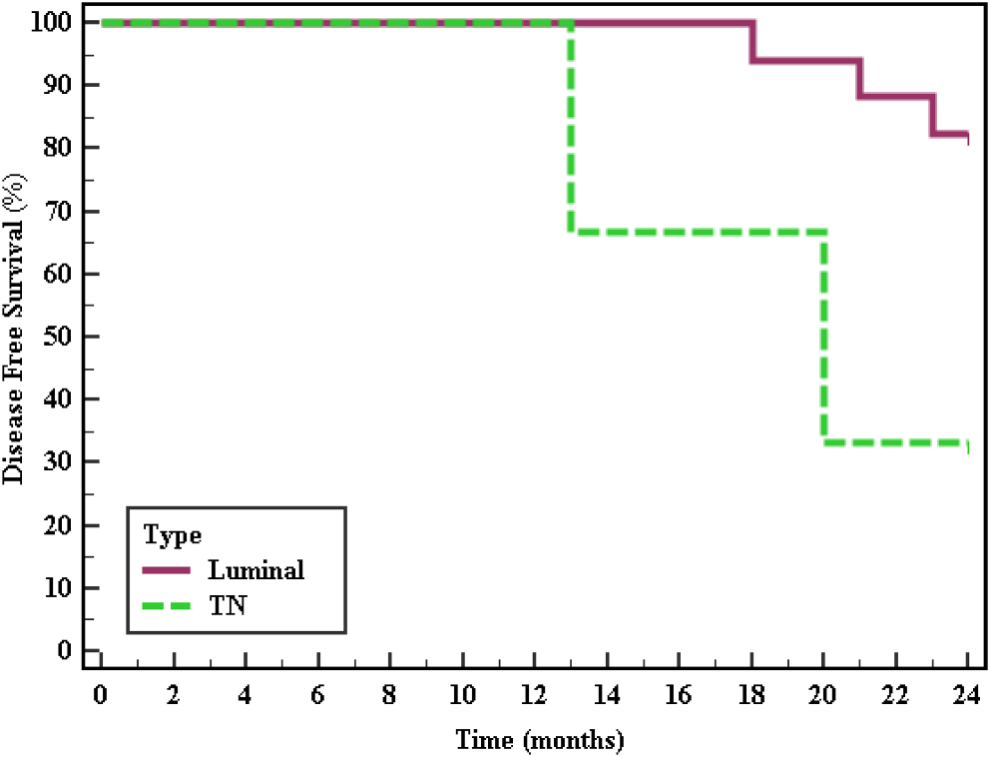
Kaplan-Meier survival curve for disease free survival with subtype (*n* = 20)

After two years of follow up, 5 patients suffered from disease related events. Two patients had local recurrence at the mastectomy scar, while another two suffered from liver metastasis and one patient had bone metastasis. Survival data analysis showed that the level of BECN1 expression has not any association with disease free survival, p: 0.944, Fig. 3; Table 6. However, when we examined molecular subtypes of patients BC, triple negative breast cancer cases had significantly lower disease free survival rate than luminal BC patients (p: 0.022), with mean DFS 19.0 months, while luminal BC patients had mean DFS of 23.41 months, Fig. 4; Table 7. On the other hand, neither stage, nor grade showed any association with DFS of the studied patients.

**Table 6:** Kaplan-Meier survival curve for disease free survival with BECN1 ratio (*n* = 17)

	Mean	% End of study	Log rank	
			χ^2^	*P*
*BECN1 T/N ratio*				
< 1	22.29	71.4	0.005	0.944
≥ 1	22.70	70.0		

**Table 7:** Kaplan-Meier survival curve for disease free survival with subtype (*n* = 20)

	Mean	% End of study	Log rank	
			χ^2^	*p*
*Subtype*				
Luminal	23.41	82.4	5.265^*^	0.022^*^
TNBC	19.0	33.3		

## Discussion

Twenty female breast cancer patients were recruited in our study and followed up for 2 years for detection of disease relapse and progression. Median age at presentation was 49 with a quarter of patients (5) below 40 years of age and 6 patients between 40–49. The age of presentation in our study is in agreement with studies in Egyptian female cohorts as reported by Azim et al. 2023 in their metanalysis. They reported that, the median age of presentation across studies was 50.4 years and about 20% were younger than 40 years (Azim et al. 2023). Screening and early detection for breast cancer remain the best strategies to manage the disease. Setting an age to start screening is equally important. With increasing technology modalities earlier detection at younger ages is possible. Given that 25% of our studied group of patients experienced breast cancer at a relatively young age raises the need for safer earlier detection of the disease and raising awareness. Some guidelines still recommend screening at older ages; the European Society for Medical Oncology (ESMO) (2019) recommends mammogram for females above 50 to 69 annually or every 2 years. Guideline also stated that regular mammogram may be offered to females 40–49 and 70–74 years of age however, more established evidence of its benefit is needed to justify its practice in those age categories, no specific screening frequency is recommended for those age groups (Cardoso et al. 2019). On the other hand, the American College of Obstetricians and Gynecologists (ACOG) (2017) recommends clinical breast examination and raising awareness in females below 40; (25–39) every 1–3 years, whereas above 40 years of age should opt to have mammograms (“Practice Bulletin Number 179: Breast Cancer Risk Assessment and Screening in Average-Risk Women,” 2017).

Autophagy process has been linked to aging where the decrease of autophagy activity correlates with dysfunctional organelles accumulation and the promotion of age-related diseases such as neurodegenerative disorders (Barbosa et al. 2018). A study by Shibata et al. 2006 on human brain found that BECN1 expression was reduced with aging (Shibata et al. 2006). On the other hand, its high levels were found to promote longevity as in a study demonstrated by Emanuele et al. 2014 on healthy centenarians (Emanuele et al. 2014). However, when we examined BECN1 expression in relation to the ages of our studied group, no correlation was found; *p* = 0.113, Table 2. In accordance with our study Li et al. 2010 reported no correlation between age and BECN1 expression on their studied cohort; median age 48.5 years (range: 40–74) (Li et al. 2010). A negative correlation was found between age and BECN1 in a study by Wang et al. 2015; however, the age group was slightly different from our study; median 53.4 years (ranging from 45 to 76) (Wang et al. 2015).

BECN1 haploinsufficiency is considered one of tumorigenesis pathways in breast cancer (Vega-Rubín-de-Celis 2019), thus it is expected to be expressed at lower levels in tumor tissues than in normal ones. In our study, we analyzed the expression of BECN1 in both tumor tissues and their corresponding adjacent normal tissues although median expression was lower in tumor tissues, it was not statistically significant, *p* = 0.463; median relative expression was 0.73 (0–8.95) in tumor tissues, while 1.02 (0.04–19.59) in adjacent normal ones, Table 3. Moreover, 10 (58.8%) of tumor tissues exhibited lower expression of BECN1 than their corresponding normal ones, Table 4.

Decreased expression of BECN1 in breast tumor tissues was also observed in several studies (Liang et al. 1999; Vega-Rubín-de-Celis 2019; Wu et al. 2012). Li et al. 2010; demonstrated that haploinsufficiency, which in part was due to aberrant BECN1 promoter methylation and copy number deletion, was the underlying cause for decreased expression (Li et al. 2010).

A statistically significant negative correlation was found between grade and BECN1 mRNA expression in the studied cases, where grade 3 demonstrated a significant lower expression than grade 2 tumors, *p* = 0.028, Table 2. Such finding denotes that BECN1 decreased expression might be associated with more aggressive tumor behavior. This observation was also reported by Wu et al. 2012 and Tang et al. 2015 (Tang et al. 2015; Wu et al. 2012).

BECN1 expression in our study was investigated in relation to ER, PR, HER2 expression. Although the level of expression was lower in ER negative, PR negative and HER2 overexpression, Table 5 the finding was not statistically significant, *p* = 0.859,0.859,0.785 respectively. The studied samples’ molecular subtypes were luminal and triple negative (TNBC). BECN1 expression was lower in TNBC however, that observation was not statistically significant as well, *p* = 0.859, Table 5. A significant association was reported by Tang et al. 2015; between lower expression of BECN1 and ER negative subtypes of breast cancer (Tang et al. 2015). The study was conducted on a much larger cohort of cases, which might explain the lack of statistical significance in our study. Cicchini et al. 2014 also reported lower expression of BECN1 mRNA in TNBC as well as poor prognosis, study was done on data from two large cohorts. (Cicchini et al. 2014).

BECN1 expression was not associated with stage, lymph node metastasis or tumor size in our study, *p* = 0.435, 0.541, 0.296, Tables 2 and 5. In consistency with our study, Wang et al. 2015 reported no association between tumor size and BECN1 expression in triple negative breast cancer using immunohistochemical staining. On the other hand, they observed high expression in association with nodal metastasis (Wang et al. 2015). The lack of association between ER, PR and nodal metastasis in our study was also reported by Li et al. 2010 study on a cohort of 20 female patients (Li et al. 2010).

A study by Yao et al. 2011 reported lower expression of BECN1 mRNA as well as protein expression (by Western blot) in breast tumor tissues than in adjacent normal ones. In accordance with our study, they found that lower expression in BECN1 (mRNA and protein levels) was associated with poor tumor differentiation. They also reported association of nodal and distant metastasis with lower BECN1 expression. However, lack of association of BECN1 expression and tumor size, PR and HER2 positivity was observed in agreement with our studied cohort (Yao et al. 2011).

The patients were followed up for a period of 2 years with thorough clinical examination and imaging where applicable and according to guidelines. 5 out 20 patients had disease relapse before 24 months of follow up. Mean DFS was found to be similar in patients with BECN1 ratio < and ≥ 1; 22.29 and 22.7 months respectively with no statistically significant difference, *p* = 0.944, Fig. 3; Table 6, a finding also reported by Wang et al. 2015 when they examined BECN1 expression and survival in 5 years follow up for TNBC patients (Wang et al. 2015). On the contrary to our finding BECN1 low expression was found to be associated with poorer prognosis in breast cancer patients in the study conducted by (Tang et al. 2015).

On the other hand, statistically significant decreased DFS was observed among patients with triple negative subtypes than luminal subtypes with mean DFS 19 months for TNBC and 23.41 months for luminal subtype, *p* = 0.022, Fig. 4; Table 7. This finding highlights the poor prognosis of TNBC and the importance for early detection and aggressive management of this disease. DFS was not correlated with stage or grade in our study; stage I, II, III: 23.75, 23, 21.67 months respectively, *p* = 0.825, while grade 2 and 3: 22.76 and 22.67 months respectively, *p* = 0.718 (data not shown). Triple negative breast cancer was repeatedly reported to have poor prognosis (Fan et al. 2023; Hennigs et al. 2016; Zagami and Carey 2022), which is consistent with our finding. Poor prognosis of TNBC is largely due to challenging therapeutic management with the lack of specific targeted therapy for this group (Yin et al. 2020).

There are several conflicting results of studies regarding BECN1 expression on protein level using immunohistochemical method. El-Guindy et al. 2023, reported that high expression of BECN1 and not lower expression is associated with poorer prognosis using cox regression model as well as its association with higher tumor grade, stage, and nodal metastasis in TNBC patients (El-Guindy et al. 2023). On the other hand, Cervantes-Díaz et al. 2022 study showed no statistically significant association between BECN1 expression and stage, tumor grade or nodal metastasis in TNBC and non TNBC (Cervantes-Díaz et al. 2022). The discordance between expression of protein and mRNA has been reported before (Kosti et al. 2016). Hence, it would be important to study the relation between BECN1 mRNA and protein expression in a larger homogenous cohort to properly guide therapeutic modalities targeting this pathway.

BECN1 expression in BC is intriguing, with potential role in future therapeutic management and prognostic workup. Our study, despite the limitation of the small sample size, showed association between decreased BECN1 expression and BC higher tumor grade, warranting further investigations on larger cohort. Further studies examining the association of BECN1 expression with response to chemotherapy would add great value to BECN1 role in BC as well as correlating its expression with other prognostic biomarkers such as Ki-67. Two years follow up data in our cohort failed to find an association between BECN1 and DFS, longer intervals of follow up might be considered in future studies. However, we observed that TNBC had a significant lower DFS than Luminal subtype in BC Egyptian female patients.

## Conclusion

Being currently the most frequently diagnosed, female breast cancer highly impacts the world; health wise as well as economically. Early detection remains the mainstay of favorable outcome. Given the high percentage of young females diagnosed with breast cancer in our study, early clinical examination for these age groups in addition to raising awareness is of utmost importance and should be emphasized in Egypt. In our study, we highlighted the potential association of BECN1 expression in breast cancer with poor tumor differentiation denoting its possible link with disease aggressiveness. However, larger cohorts are required to demonstrate its impact on overall survival and disease-free survival with longer interval of follow up. Triple negative breast cancer subtype remains by far associated with less favorable prognosis, urging the need for better therapeutic solutions and deeper molecular characterization.

## Abbreviations

ACOG: American college of obstetricians and gynecologists
BC: Breast cancer
BECN1: Beclin-1
cDNA: complementary deoxyribonucleic acid
DFS: Disease free survival
ER: Estrogen receptor
ESMO: European society for medical oncology
FFPE: Formalin fixed paraffin embedded
FISH: Fluorescence in situ hybridization
GAPDH: Glyceraldehyde-3-phosphate dehydrogenase
H&E stain: Hematoxylin and eosin stain
HER2: Human epidermal growth factor receptor 2
IDC: Invasive ductal carcinoma
IHC: Immunohistochemistry
mRNA: messenger ribonucleic acid
mTOR: mammalian target of rapamycin
NST: No special type
PIK3C3: Phosphatidylinositol 3-kinase class III
PR: Progesterone receptor
RNA: Ribonucleic acid
SD: Standard deviation
TNBC: Tripple Negative breast cancer
ULK complex: Unc51-like kinase complex
VPS34: Vacuolar protein sorting 34
